# Transdermal Delivery of Capsaicin Nanoemulgel: Optimization, Skin Permeation and *In Vivo* Activity against Diabetic Neuropathy

**DOI:** 10.34172/apb.2022.080

**Published:** 2021-10-09

**Authors:** May Saab, Karim Raafat, Hoda El-Maradny

**Affiliations:** ^1^Department of Pharmaceutical Technology, Faculty of Pharmacy, Beirut Arab University, Beirut 11072809, Lebanon.; ^2^Department of Pharmacognosy and Natural Products, Faculty of Pharmacy, Pharos University in Alexandria, Alexandria, Egypt.; ^3^Department of Industrial Pharmacy, Faculty of Pharmacy, Alexandria University, Alexandria, Egypt.

**Keywords:** Capsaicin, Eucalyptus oil, Nanoemulsion, Transdermal, Diabetic neuropathy, Pain

## Abstract

***Purpose:*** Diabetic somatic neuropathy is one of the most prevalent complications in type 1 diabetes mellitus (T1D). Many treatments were investigated to alleviate the pain associated with this condition. Capsaicin is a naturally occurring lipophilic alkaloid that proved to be an effective and safe treatment of chronic painful disorders. Despite the known therapeutic benefits of capsaicin, the conventional topical formulations have limited bioavailability. Therefore, the current study aims to develop capsaicin nanoemulgel to increase skin permeation and enhance its activity against neuropathic pain.

***Methods:*** Low-energy emulsification method was used to prepare nanoemulsions, using eucalyptus oil as the oily phase, Tween 80 as a surfactant, propylene glycol, ethanol and isopropyl alcohol as co-surfactants. Pseudo-ternary phase diagrams were constructed to investigate and optimize the formulation. Subsequently, the optimum formulation was formulated as a nanoemulgel and investigated for, skin permeation using Franz diffusion cell, and diabetic neuropathy (DN) management using alloxan-induced diabetic mice.

***Results:*** The selected nanoemulsion containing 0.05% capsaicin is composed of 8 % oil, 24 % S _mix_ (Tween 80: isopropyl alcohol 2:1 w/w) and 68 % water. It is characterized by nanosized globules (28.15 ± 0.24 nm) with a relatively low polydispersity index (0.27 ± 0.05). The nanoemulgel revealed *circa* 4-fold increase in capsaicin cumulative permeation when compared to the conventional gel, and an improvement in its antinociceptive properties was observed in the treated diabetic mice (*P* < 0.05).

***Conclusion:*** The selected capsaicin nanoemulgel would be a promising transdermal formulation that may alleviate diabetic neuropathy in T1D patients.

## Introduction

Type 1 diabetes mellitus (T1D) is a widespread disease that is usually associated with many chronic complications.^1^ Diabetic neuropathy (DN) is one of the most prevalent complications, which is manifested by chronic painful tactile allodynia and hyperalgesia.^2^ In sight of the lack of new and more safe drugs for efficient amelioration of DN, medicinal plant extracts or herbal Phyto-Pharmaceuticals can be an alternative resource for modern-medicine in the development of an effective treatment.^3^

In the last two decades, capsaicin, has been investigated as a potential therapeutic agent for many diseases, including cancer,^4^ cardiovascular diseases,^5,6^ and predominantly many painful conditions.^7^ This naturally occurring lipophilic alkaloid (vanilloid) that has been extracted from capsicum fruits proved to an effective treatment of chronic and inflammatory pain disorders, such as rheumatoid arthritis and osteoarthritis,^8^ DN and musculoskeletal pain disorders,^9^ and alleviation of post-operative pain.^10^ The mechanism of its analgesic effect lies in the activation of the transient receptor potential vanilloid type 1 receptors that are mainly expressed by sensory neurons. Stimulation of these receptors causes depletion of vasoactive inflammatory neuropeptides (substance P) from C-fiber nerve terminals, resulting in neuronal desensitization.^11^ Additionally, its anti-inflammatory property is exhibited by inhibiting the activity of some pro-inflammatory mediators such as PEG2, inducible nitric oxide synthase (iNOS) and cyclooxygenase-2 (COX-2).^12^ Despite the proven efficacy of capsaicin in different painful conditions, its clinical use intravenously was not advocated, as it exhibited a short half-life of 7.06 min. Its oral administration was also limited due to many drawbacks, including its first pass metabolism, pungency and non-compliance.^13,14^

Nanotechnology-based transdermal delivery has received considerable interest in the pharmaceutical industry as it is advantageous over oral delivery system; it could avoid the first-pass effect, provide better patient compliance, improve drug solubility and attain better bioavailability.^15^

Many nanocarriers were investigated and proved to be promising transdermal drug delivery systems; these include solid lipid nanoparticles,^16^ nanostructured lipid carriers,^17^ niosomes,^18^ nanoemulsions,^19^ and liposomes.^20^ Nevertheless, nanoemulsions are considered as one of the most favorable nanocarriers for the transdermal delivery of hydrophobic drugs. These systems are easily formulated, and are characterized by their clarity and high stability that maintain the drug in a molecularly dispersed state, leading to an enhanced skin permeability and improved bioavailability.^21,22^ Nanoemulsions can provide better capsaicin bioavailability and may also minimize skin irritation, when compared to conventional formulations such as creams, lotions and gels.^23,24^

Few studies about nanoemulsion-based transdermal delivery of capsaicin have been found. These include nanoemulsions prepared using different oily phases, such as isopropyl myristate,^25^ oleoresin capsicum,^26^ and olive oil.^19^

In the present work, capsaicin transdermal nanoemulsions were formulated for the treatment of DN using eucalyptus oil as the oily phase. The selection of eucalyptus oil was based on its spontaneous emulsification into nanosized globules by low energy method and its preservative effect,^27,28^ that would be advantageous over previously investigated oils. The aim of the current study was to prepare and optimize capsaicin nanoemulsions and investigate its skin permeability and in-vivo effectiveness against DN, endeavoring for an improvement of painful tactile allodynia and hyperalgesia conditions.

## Materials and Methods

###  Materials

 Capsaicin, Eucalyptus oil, Carbopol 940, triethanolamine, McCoy 5A medium and alloxan were all purchased from Sigma-Aldrich, USA. Ethanol (≥ 99.5%), isopropyl alcohol (≥ 99.7%), propylene glycol and Tween 80 were obtained from Fluka, Germany.

###  Construction of pseudo-ternary phase diagrams

Prior to drug incorporation, different pseudo-ternary phase diagrams were constructed using water titration method.^29^ The diagrams consisted of eucalyptus oil, Tween 80 surfactant/co-surfactant mixture (S_mix_) and water. Eucalyptus oil was mixed with Tween 80 or with a mixture of Tween 80 and co-surfactant mixture (S_mix_), at different ratios ranging from 1:9 to 9:1 (w/w). All mixtures were titrated dropwise with water with gentle stirring until turbidity or milky appearance was detected. Clear and easily flowable formulations were considered as part of the nanoemulsion region in the phase diagram.

 Three types of co-surfactants were investigated, namely, propylene glycol, ethanol and isopropyl alcohol, such that the ratio of Tween 80 to co-surfactant was fixed at 1:1 w/w. The best co-surfactant was then selected based on the largest nanoemulsion existence area in the pseudo-ternary phase diagram, and further optimization was conducted by using different ratios of Tween 80 and the selected co-surfactant (1:1, 1:2, 2:1 and 3:1 w/w).

###  Droplet size and polydispersity index

 The average droplet size and polydispersity index of nanoemulsions were determined using Zetasizer (Malvern Zetasizer Nano-ZS90, Worcestershire, UK). All measurements were performed at an angle of 90° at 25°C.

###  Transmission electron microscope (TEM)

Morphology of the optimized formulation was investigated using Morgagni 268D transmission electron microscope (FEI Company, the Netherlands). Samples were placed on a carbon-coated grid and treated with one drop of phosphotungstic acid (2%). Subsequently, the treated sample was subjected to air drying and covered with a slip prior to observation under TEM.^30^

###  Viscosity and conductivity of the nanoemulsion

 The viscosity of the nanoemulsion was determined undiluted using Brookfield viscometer (DV-II + Pro Viscometer, Bohlin Visco 88, Malvern, UK), where samples (30 mL) were allowed to equilibrate for 5 minutes prior to measurement.

 Electrical conductivity was measured using a conductivity meter (EC tester II, USA) at room temperature. All measurements were conducted in triplicates.

###  Stability study of capsaicin nanoemulsion

Accelerated stability tests were conducted for the optimum capsaicin nanoemulsion. Tests were followed by visual observation of the formulation to detect any turbidity or phase separation, followed by spectrophotometric measurement of its % transmittance at λ_max_ 600 nm.^31^

First, the optimized nanoemulsions were centrifuged (5000 rpm) for 30 minutes at 25°C. Once passed the centrifugation test, samples were subjected to three consecutive heating-cooling cycles; nanoemulsions were kept at 40°C for 48 hours and subsequently at 25°C for the same period of time.^32^ Furthermore, three successive freeze thaw cycles were carried out by freezing the nanoemulsion at -21°C for 2 days, followed by thawing the sample at 25°C for another two days.^30^

Additionally, long term stability study was carried out, where nanoemulsions were stored for 6 months at 25°C, 4°C and 40°C.^19^ All tests were performed in triplicates.

###  Formulation of nanoemulgel

The nanoemulgel was prepared by mixing the selected nanoemulsion with the gel base. The gel was prepared according to the method of Harwansh et al with slight modification.^33^ Briefly, 1% gel was prepared by dispersing 1 g of Carbopol 940 in 100 mL deionized water and allowed to swell for 24 hours at room temperature. After complete swelling, 10 g of propylene glycol and isopropyl alcohol were added and mixed using a magnetic stirrer. Subsequently, the prepared gel was added slowly and in equal amount to the nanoemulsion while stirring (100 rpm). Lastly, few drops of triethanolamine were added and mixed to obtain an emulgel with neutral pH.

###  Rheological properties of nanoemulgel

The viscosity of nanoemulgel was determined using a Brookfield viscometer having cone and plate configuration (Bohlin Visco 88, Malvern, UK). Rheological study was conducted by applying a shear rate ranging beTween 1 and 100 s^-1^ at room temperature. The consistency index (K) and flow index (n) were calculated from the power law equation as follows:


Eq.(1) $$\tau =Kr^{n}$$

where *τ* is the shear stress and *r* are the shear rate.

###  Spreadability of nanoemulgel

Spreading property of the nanoemulgel was investigated as previously reported by Arora et al.^34^ A specified amount of nanoemulgel was weighed (500 mg) and placed in the center of a glass plate within a marked circumference of 1 cm in diameter for 1 minute. A second glass plate was then placed over the first one so that the nanoemulgel is sandwiched beTween the two plates. Consequently, the increase in diameter resulting from the added weight was recorded, and the spreadability (S) was calculated by using the following equation:


Eq.(2) $$S=\frac{m.\Delta d}{t}$$

where, *m* is the added weight in g, *Δd* is the increase in diameter in cm and *t* is the time in seconds.

###  Skin permeation study

**Figure 1:**
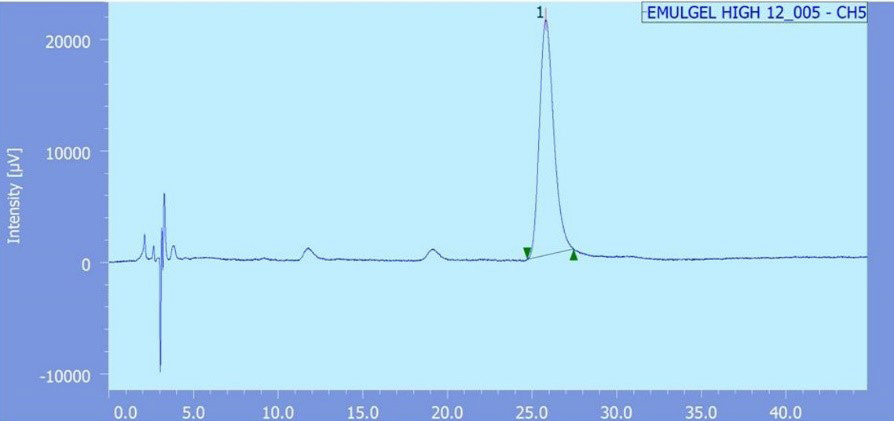
Chromatogram of permeated capsaicin

Drug permeation through the skin was investigated *ex vivo* using a vertical Franz diffusion cell as previously described by Arora et al, with some modifications.^34^ Dorsal skin of Wistar rat was excised after hair removal, and the excess fatty and connective tissues were carefully removed using a scalpel. Subsequently, the excised skin was washed with normal saline and kept refrigerated at 4ºC in McCoy’s 5A medium to preserve the viability of the skin until its use.^35^ Skin was mounted beTween the donor and receptor compartments (diffusion area = 2 cm^2^), such that the stratum corneum was facing upward in the direction of the donor compartment. The receptor compartment was filled with 25 mL of phosphate buffer (pH 7.4)/ethanol solution (1:1 v/v), that was maintained at 37ºC and constantly stirred at 100 rpm. One g of the formulation was placed over the skin in the donor compartment. At specified time intervals (0.5, 1, 2, 3, 4, 5, 6, 8, 10, 12 and 24 hours), 200 µL samples were withdrawn from the receiver compartment and instantly compensated with fresh medium. Subsequently, samples were analyzed by reverse phase high performance liquid chromatography method (RP-HPLC) as reported earlier.^36^ Briefly, the mobile phase consisted of a mixture of methanol and water at a ratio 3:2 v/v, flowing at a rate of 0.6 ml/minute through the column (C18, waters, Sunfire, 5 μm, 4.6*250 mm, Ireland). Samples’ peaks were detected spectrophotometrically at λ_max_ 280 nm using UV detector (Waters 2487 Dual λ absorbance, Waters corporation, Milford, MA) (Figure 1). All recorded measurements were an average of triplicate.

The cumulative amount of capsaicin permeated per unit area of skin (Q/S) was plotted against time t. Then, the flux or skin permeation rate at steady state (Jss) was computed from the slope of the linear segment of the plot as follows^26,37^:


Eq.(3) $$J_{ss}=\frac{\Delta Q_{t}}{\Delta_{t}\times S}$$

Subsequently, enhancement ratio (Er) was calculated from the following equation:^37^


Eq.(4) $$E_{r}=\frac{J_{ss}\;of\;nanoemulgel}{J_{ss}\;of\;conventional\;gel}$$

###  In-vivo investigation

Male Swiss-Webster mice weighing beTween 20 and 30 g have been housed in cages with 12-hour light/dark cycles for seven consecutive days prior to the experiment as described earlier.^38^

Briefly, sterile intraperitoneal (IP) injection of alloxan (dissolved in 0.9 % NaCl) was administered to mice at a dose of 180 mg/kg every 48 hours to induce T1D. Blood glucose level (BGL) and HbA1c value have been measured using Accu-Chek Active TM glucometers (Roche, USA) and HbA1c micro column method (Analyticon, Germany), respectively. The animals were considered diabetic if BGL and HbA1c were above 200 mg/dL and 8 %, respectively.^39^

Following 6 weeks of induction of T1D, DN success rate was *circa* 90% as described in an earlier study.^40^ Different complementary tests have been carried out to assess DN management throughout a period of 8 weeks. The tests were conducted on different groups of mice (Table 1). The response to each test following the intended treatment was monitored and recorded on weekly basis.

**Table 1:** Groups of animals tested with different formulations

**Group code**	**n**	**Description**	**Tested formulation**
NORM	6	Normal mice	None
DIA + Placebo	6	Diabetic mice	Placebo gel
TRA 10 mg/kg	6	Diabetic mice	Tramadol 10 mg/kg
DIA + Conv	6	Diabetic mice	Capsaicin conventional gel
DIA + Nano	6	Diabetic mice	Capsaicin nanoemulgel

####  Hot plate latency test

Hot plate latency test was carried out to evaluate the management of thermal hyperalgesia as previously stated.^39^ In short, mice were positioned on the hot plate apparatus (Ugo Basile, Italy) with an adapted temperature of 55 ± 0.1°C. The index of thermal nociceptive threshold was reflected by the time taken for initial hind paw withdrawal or licking. Maintaining a cut-off duration of 30 seconds has been attempted to prevent tissue-damage.^41^

####  Tail flick latency test

Alongside the hot plate test, tail flick latency experiment was carried out as a supplementary test to assess the amelioration of thermal hyperalgesia.^39^ Briefly, mice were placed in the tail-flick apparatus (Hugo Sachs Electronic, Germany) and the time elapsed from the onset of radiant heat till tail’s withdrawal was recorded with a 10 seconds cut-off to avoid tissue-damage.^41^

####  Von Frey filaments test

Von Frey filaments test was carried out to measure tactile allodynia as described earlier.^38^ Concisely, mice were placed in an elevated inverted cage having a wire mesh floor and allowed to adapt for 10 minutes. Subsequently, Von Frey filaments (Opti Hair^TM^, Marstock Nervtest^TM^, Germany) of different intensities (0.5-45.3 g) were used and paw withdrawal after filament removal was recorded as a positive response to the mechanical stimuli.

## Results and Discussion

###  Screening of co-surfactants

**Figure 2:**
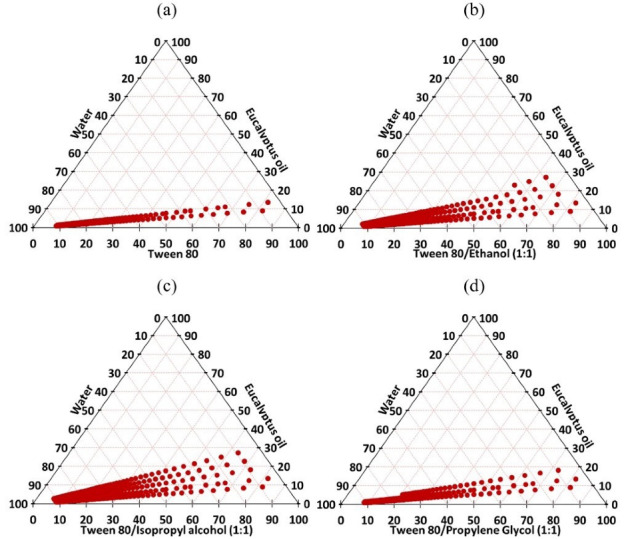
Pseudo-ternary phase diagrams of eucalyptus oil, water and Tween 80 (a) and eucalyptus oil, water and Tween 80 /cosurfactant (S_mix_) at 1:1 ratio (b, c and d)

At the beginning, a pseudo-ternary phase diagram was constructed using eucalyptus oil as the oily phase and Tween 80 as the surfactant. Surfactants with an HLB ranging be Tween 9 and 20 are essential for the formation of oil in water nanoemulsions. Therefore, Tween 80 (HLB 15) was selected as a suitable surfactant for the emulsification of eucalyptus oil.^28,29^ Despite the low energy needed to prepare nanoemulsions and its thermodynamically spontaneous formation, a careful observation was required to avoid the selection of metastable systems.^42^ The nanoemulsion zone was depicted in the phase diagram (Figure 2a). The size of this region indicates the nanoemulsification efficacy of the system. The nanoemulsion area was not sufficiently large, indicating that the surfactant alone was not able to efficiently reduce the interfacial tension at the o/w interface. Therefore, the addition of co-surfactants to the system was advocated.

**Figure 3:**
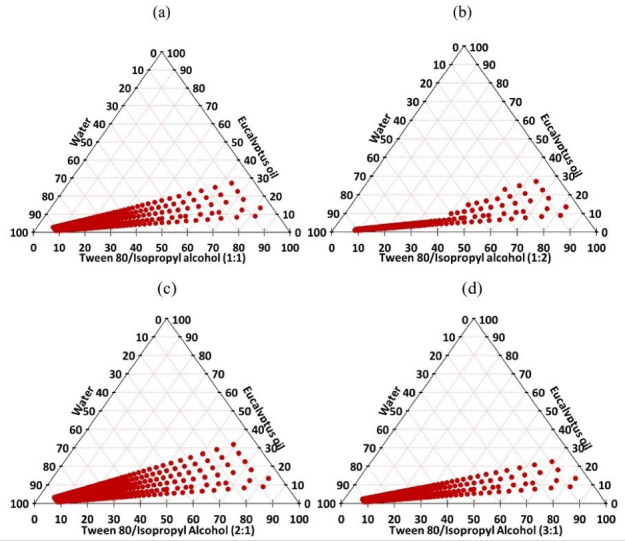
Pseudo-ternary phase diagrams of eucalyptus oil, water and Tween 80:isopropyl alcohol (S_mix_) at different ratios; 1:1 (a), 1:2 (b), 2:1 (c) and 3:1 (d)

Alcohols with short and medium chain hydrocarbons are usually incorporated as co-surfactants during the formulation of nanoemulsions to lower the concentration of surfactants.^43^ Additionally, the partitioning of these co-surfactants be Tween oily and aqueous phases can decrease the interfacial tension and permit higher miscibility of the two phases.^44^ Hence, ethanol, isopropyl alcohol and propylene glycol were chosen as co-surfactants and mixed at a fixed ratio with Tween 80 (1:1 w/w). The evaluation of co-surfactants was mainly dependent on the area of the formed nanoemulsion illustrated in the pseudo-ternary phase diagram (Figures 2b-2d). It was found that the addition of cosurfactants at S_mix_ ratio 1:1 w/w resulted in an expansion in the nanoemulsion zone. This could be attributed to a further reduction of the interfacial tension and an increase in the dispersion entropy.^34^ Isopropyl alcohol provided the largest nanoemulsion region, followed by ethanol and propylene glycol. When compared to propylene glycol, the lower number of hydroxyl groups in isopropyl alcohol and ethanol would be a contributing factor in more stable nanoemulsions and larger zones. However, the superiority of isopropyl alcohol over ethanol as co-surfactant could be explained by the longer hydrocarbon chain of the former.^44^ Based on these results, isopropyl alcohol was selected as the best co-surfactant. The variation in S_mix_ mass ratio is a key factor that would influence the size of the nanoemulsion zone.^45^ Therefore, further optimization was conducted by constructing phase diagrams using different S_mix_ (Tween 80: isopropyl alcohol) ratios (Figure 3). As the concentration of isopropyl alcohol increased (i.e. S_mix_ 1:2), a decrease in the nanoemulsion zone was detected when compared to S_mix_ 1:1. On the other hand, an increase in surfactant concentration (i.e. S_mix_ 2:1) led to an expansion in the nanoemulsion zone when compared to that of S_mix_ 1:1. Such discrepancy in phase behavior would be explained by the variation in the packing of S_mix_ at the o/w interface.^44^ However, a further increase in surfactant concentration (i.e. S_mix_ 3:1) resulted in a decrease in the nanoemulsion area, thereby, indication that S_mix_ 2:1 was the best ratio for optimum emulsification.

###  Selection and characterization of the optimum formulation

Distinct and separate points in the nanoemulsion zone of the optimized diagram were selected, and formulations corresponding to each point were prepared. Subsequently, the formulated emulsions were investigated in an attempt to select the optimal formulation for drug incorporation (Table 2). A decrease in the droplet size (DS) and polydispersity index (PDI) value was observed when the oil concentration decreased. This was in alignment with the findings reported in previous studies, where researchers have related the reduction of the oily globule size and PDI of the prepared nanoemulsions to the decrease in oil concentration.^34,44^

**Table 2:** Characterization of nanoemulsions at different oil:S_mix_ ratios

**Oil: S**_mix_ ** (W/W)**	**Code**	**Oil (%)**	**S**_mix_^*^ **(%)**	**Water (%)**	**DS (nm)**	**PDI**
1:9	N1	9	81	10	672 ± 33.24	0.75 ± 0.15
	N2	8	72	20	232 ± 9.28	0.68 ± 0.10
	N3	6	54	40	62.51 ± 5.73	0.62 ± 0.12
	N4	4	36	60	20.82 ± 3.64	0.59 ± 0.14
	N5	2	18	80	12.9 ± 0.26	0.21 ± 0.03
	N6	1	9	90	11.6 ± 0.31	0.20 ± 0.04
1.5:8.5	N7	10	56.7	33.3	142.5 ± 10.26	0.50 ± 0.09
	N8	8	45.6	46.4	92.3 ± 8.97	0.47 ± 0.10
	N9	6	34.2	59.8	65.5 ± 5.62	0.42 ± 0.09
	N10	4	22.8	73.2	41.3 ± 3.76	0.42 ± 0.11
	N11	2	11.4	86.6	24.2 ± 2.95	0.40 ± 0.13
	N12	1	5.7	93.3	20.7 ± 1.77	0.35 ± 0.09
2:8	N13	15	60	25	321.2 ± 13.54	0.70 ± 0.11
	N14	10	40	50	286.7 ± 9.41	0.69 ± 0.08
	N15	8	32	60	42.3 ± 1.83	0.54 ± 0.07
	N16	6	24	70	31.6 ± 1.44	0.42 ± 0.08
	N17	4	16	80	15.3 ± 0.22	0.28 ± 0.02
	N18	2	8	90	14.5 ± 0.16	0.13 ± 0.01
	N19	1	4	95	12.2 ± 0.13	0.11 ± 0.01
2.5:7.5	N20	20	60	20	146.6 ± 6.14	0.36 ± 0.05
	N21	15	45	40	87.87 ± 3.92	0.44 ± 0.07
	N22	10	30	60	25.3 ± 1.73	0.40 ± 0.08
	N23	8	24	68	23.8 ± 0.15	0.24 ± 0.02
	N24	6	18	76	23.2 ± 0.12	0.26 ± 0.01
	N25	4	12	84	21.5 ± 0.11	0.19 ± 0.02
	N26	2	8	90	16.4 ± 0.22	0.13 ± 0.01
	N27	1	4	95	16.3 ± 0.31	0.14 ± 0.01
3:7	N28	25	58.25	16.75	374.5 ± 15.72	0.68 ± 0.13
	N29	20	46.6	33.4	113.3 ± 6.53	0.64 ± 0.11
	N30	15	34.9	50.1	101.4 ± 5.64	0.52 ± 0.09
	N31	10	23.3	66.7	58.48 ± 4.32	0.47 ± 0.12
	N32	8	18.6	73.4	32.31 ± 2.96	0.44 ± 0.08
	N33	6	14	80	29.6 ± 1.82	0.38 ± 0.11
	N34	4	9.3	86.7	26.4 ± 0.09	0.30 ± 0.04
	N35	2	4.7	93.3	25.8 ± 0.12	0.31 ± 0.05
	N36	1	2.3	96.7	25.6 ± 0.08	0.29 ± 0.02
3.5:6.5	N37	30	55.8	14.2	464.5 ± 21.25	0.76 ± 0.13
	N38	25	46.5	28.5	409.2 ± 18.45	0.71 ± 0.14
	N39	20	37.2	42.8	255.6 ± 14.11	0.67 ± 0.09
	N40	15	27.9	57.1	104.8 ± 6.72	0.56 ± 0.08
	N41	10	18.6	71.4	65.1 ± 3.87	0.52 ± 0.06
	N42	8	14.9	77.1	41.7 ± 3.64	0.45 ± 0.11
	N43	6	11.2	82.8	32.8 ± 2.39	0.40 ± 0.07
	N44	4	7.4	88.6	29.5 ± 1.25	0.33 ± 0.04
	N45	2	3.7	94.3	24.1 ± 1.32	0.34 ± 0.05
	N46	1	1.9	97.1	23.4 ± 0.73	0.28 ± 0.03

*S_mix_ consisted of Tween: isopropyl alcohol at a ratio 2:1 w/w, respectively.

The selection of the candidate formulations for drug incorporation was based on the globule size (< 100 nm) and its uniformity (PDI < 0.3).^26^ Small DS and PDI values were detected in N5, N6, N17, N18. N19, N23, N24, N25, N26 and N27 formulations (Table 2). Consequently, capsaicin (0.1% w/w) was incorporated in the aforementioned formulations by dissolving it in the oil phase prior to emulsification. Among the candidate formulations, N23 and N24 have succeeded in forming nanoemulsions, whereas other formulations were milky in appearance.

An increase in DS and PDI of the medicated N23 and N24 formulations was noticed, yet, the change in these characteristics was less pronounced in N23 (DS 28.15 ± 0.24 nm and PDI 0.27 ± 0.05) when compared to N24 (DS 45.62 ± 1.41 nm and PDI 0.39 ± 0.11). This could be explained by the higher content of the oily phase in the former (8 %) resulting in an enhanced solubilization of capsaicin. In other words, the lower the concentration of capsaicin within the oily phase, the smaller would be its influence on DS. In a previous work, the addition of capsaicin in olive oil nanoemulsion did not affect considerably the globule size of the latter, as this would be attributed to the different nature of the oil and its solubilizing efficiency.^19^ As a result, N23 comprising 8 % oily phase with a 2.5:7.5 (w/w) oil to S_mix_ ratio was chosen as the optimum formulation.

The viscosity of the selected nanoemulsion was low (11.5 ± 0.23 mPa·s), which was not appropriate for topical application, hence, justifying the need for an emulgel formulation. Additionally, the conductivity of the formulation was high (76.6 ± 0.11 µS/cm), thus, confirming that the nanoemulsion is of O/W type, as reported in previous studies.^34,46^

All the stability tests that were performed on the optimized formulation indicated a stable nanoemulsion as no turbidity or phase separation was observed and all the characteristics of the formulation did not change considerably (Table 3).

**Table 3:** Stability study of the optimized nanoemulsion

**Stability tests**	**DS (nm)**	**PDI**	**% Transmittance**	**Appearance**
Prior to stability testing	28.15 ± 1.21	0.27 ± 0.05	99.72 ± 0.25	Transparent
After centrifugation	28.43 ± 0.91	0.27 ± 0.06	97.34 ± 1.31	Transparent
After heating-cooling cycles	31.25 ± 0.87	0.28 ± 0.06	96.15 ± 0.76	Transparent
After freeze-thawing cycles	34.38 ± 1.34	0.29 ± 0.04	95.32 ± 1.98	Transparent
Long term stability (4˚C)	32.41 ± 0.62	0.28 ± 0.05	98.86 ± 1.54	Transparent
Long term stability (25˚C)	29.83 ± 0.87	0.30 ± 0.03	95.44 ± 2.12	Transparent
Long term stability (40˚C)	39.58 ± 1.19	0.31 ± 0.04	88.25 ± 2.75	Transparent

###  Characterization of the prepared nanoemulgel

 Nanoemulgel was prepared based on the optimum medicated formulation (N23), where the final concentration of capsaicin was 0.05%.

**Figure 4:**
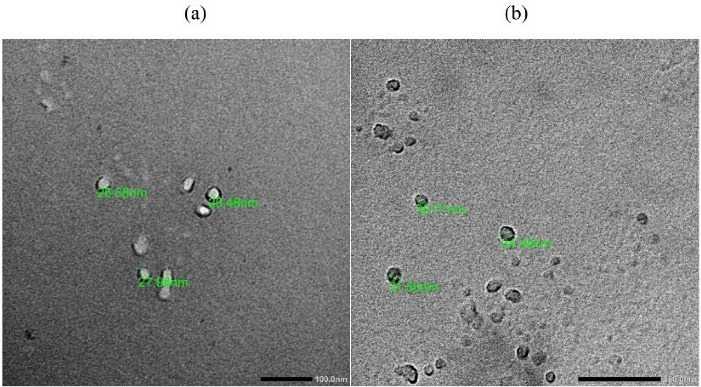
TEM images of capsaicin nanoemulsion (a) and capsaicin nanoemulgel (b)

The size and morphology of emulsion droplets in capsaicin nanoemulgel was examined under TEM and compared with that of its corresponding nanoemulsion (Figure 4). Emulsion droplets in both nanoemulsion and nanoemulgel were spherical in shape and within a nanosize range, yet, a marginal increase in the size of emulsion droplets was observed by TEM when compared to that recorded by Zetasizer. This would be explained by the treatment procedure of the sample that preceded its observation under TEM.^19^ Emulsion droplets did not coalesce after gel incorporation indicating its physical stability against Oswald ripening which was in alignment with earlier reports.^47,48^

###  Rheological behavior and spreadability of nanoemulgel

**Figure 5:**
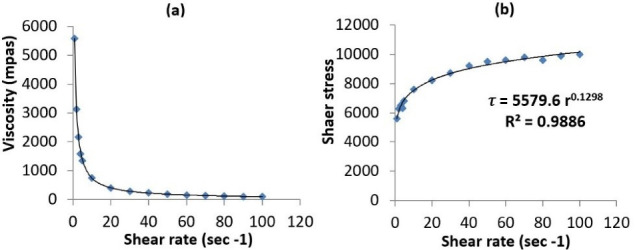
Rheograms of capsaicin nanoemulgel; viscosity vs shear rate (a), and shear stress vs shear rate (b)

Rheological behavior of the prepared nanoemulgel is an imperative property that substantially reflects its consistency, flowability and drug release. The relationship be Tween the viscosity and shear rate is graphically illustrated in Figure 5a. The rheogram shows a decrease in viscosity with an increase in the shear rate, until reaching a plateau. This would suggest a pseudoplastic behavior of the nanoemulgel.^49^ The relationship be Tween the shear stress and shear rate was also determined (Figure 5b) to compute the consistency index (K) and the flow index (n).^50,51^ K represents the apparent viscosity of the gel at 1 s^-1^ shear rate, whereas, n determines the deviation of the gel from Newtonian behavior. If the value of n is equal to 1, this indicates a Newtonian flow. When n value exceeds 1, the gel is characterized by a dilatant flow. On the other hand, a value of less than 1 implies a pseudoplastic flow. The consistency index of the nanoemulgel was found to be 5580 mPa, whereas the flow index value was 0.13, thus confirming the suggested pseudoplastic behavior. Such pseudoplasticity indicates that the nanoemulgel would need some force to be expelled from the tube.^34^

Furthermore, spreadability of nanoemulgel has been examined as it is considered an important test to investigate the maximum slip and drag of transdermal formulations, that would reflect its ease of application.^52^ The spreadability was found to be 6.73 ± 0.12 g.cm.s^-1^, thereby, indicating an easily spreadable formulation.^34^

###  Skin permeation study

**Figure 6:**
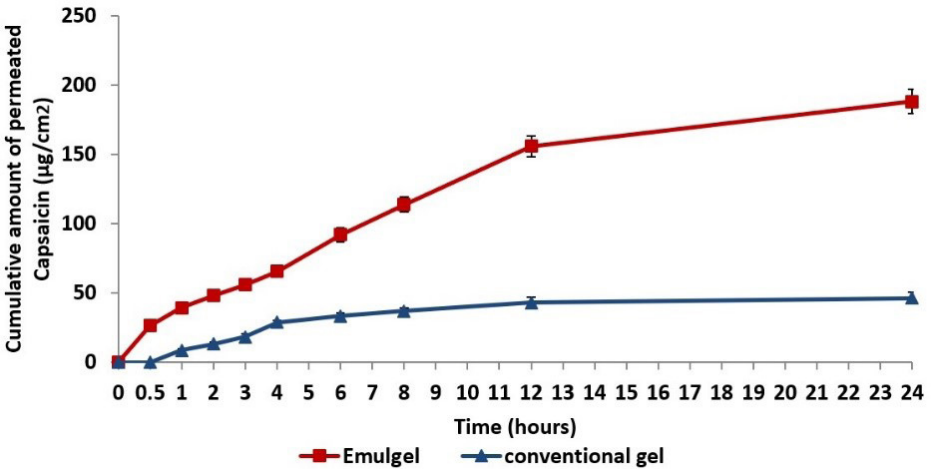
*In vitro* capsaicin permeation profile through rat skin per unit area from conventional gel and nanoemulgel (mean ± standard deviation, n = 3)

The main barrier for drug permeation through the skin is the stratum corneum layer. Unlike hydrophilic substances, lipophilic ones can easily penetrate the lipid domains in stratum corneum, yet, they have some limitations to reach the domains that have hydrophilic aspects.^53^ The presence of capsaicin molecules in a solubilized state within the nanosized eucalyptus oil droplets, and the ability of Tween 80 to fluidize the lipids within the stratum corneum and disrupt corneocytes, may contribute in enhancing drug permeation.^25,54^ In addition, the selected co-surfactant (isopropyl alcohol) may play a role in the improvement of drug absorption, as it has the ability to enhance the fluidity at the liquid-liquid interface, and may also act as a permeation enhancer by reversibly altering the physicochemical nature of the stratum corneum.^54^ Therefore, the formulated nanoemulgel would facilitate capsaicin permeation through both hydrophilic and hydrophobic layers of the skin to maximize drug absorption. To assess permeation ability of the optimized formulation, an *ex vivo* skin permeation study was conducted using Franz diffusion cell. Since capsaicin is water insoluble compound that may not easily partition into the receptor aqueous medium at in-vitro level, a solubilizing mixture of phosphate buffer and ethanol (1:1) was used.^25,36^ The permeation profiles of capsaicin nanoemulgel and conventional gel are illustrated in Figure 6. It was observed that capsaicin nanoemulgel revealed a greater and faster skin permeation than the conventional gel; the cumulative amount of capsaicin permeated at the end of 24 hours was found to be 188.12 µg/cm^2^ and 46.38 µg/cm^2^ for nanoemulgel and conventional gel, respectively. Moreover, the permeation flux of capsaicin from the nanoemulgel (0.19 µg/cm^2^.s^-1^) was superior to that of conventional gel (0.11 µg/cm^2^.s^-1^), resulting in an enhancement ratio of 1.73, thus, confirming the enhanced transdermal delivery of capsaicin in the former. Such finding is explained by the alteration of the lipophilic barrier and the hydrophilic pathways of the skin through synergistic interaction of the oil and surfactant/co-surfactant mixture of the nanoemulsion with the epidermal layers of the skin.^55,56^

###  Diabetic neuropathy management

**Figure 7:**
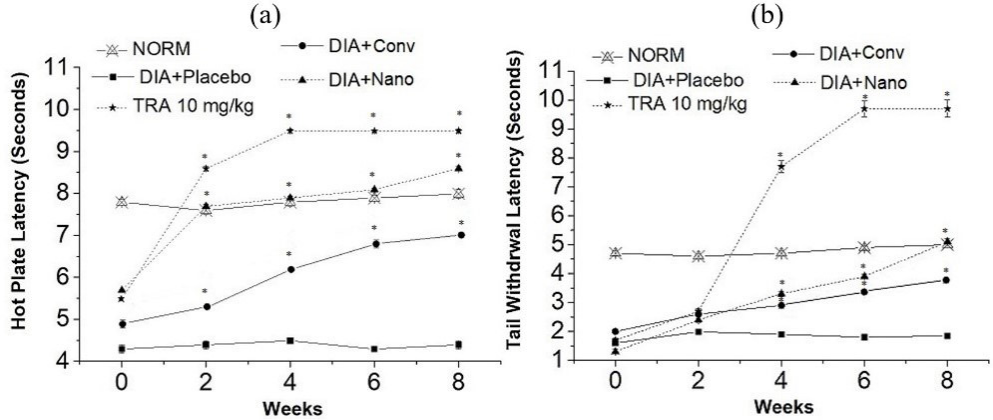
The effect of capsaicin conventional gel, capsaicin nanoemulgel, and Tramadol (TRA) 10 mg/kg on hot plate latency (a), and tail withdrawal latency (b), in neuropathic model in alloxan-induced diabetic mice (* *P* < 0.05 significant from the placebo treated animals)

The treatment of alloxan-induced diabetic mice with capsaicin nanoemulgel has markedly improved the thermal latency (Figure 7a and 7b). Upon application of the nanoemulgel, hot-plate and tail withdrawal latency was extended significantly (*P* < 0.05) by 2.0 and 2.8 folds, respectively, in the 8th week when compared to the placebo treated mice. On the other hand, treatment with conventional capsaicin gel resulted in less remarkable improvement in thermal tests with an increase by 1.6 and 2.0 folds, respectively. Moreover, Capsaicin nanoemulgel was superior to the conventional gel in ameliorating mechanical allodynia (Table 4); nanoemulgel treated group revealed a significant amelioration by 13.2 folds (*P* < 0.05) in the 8th week when compared to the placebo treated animals, hence, approaching the improvement that has been recorded by tramadol treated group. These findings can be attributed to the successful transdermal delivery of capsaicin from nanoemulgel as previously confirmed in the skin permeation study.

**Table 4:** The effect of capsaicin conventional gel, capsaicin nanoemulgel, and tramadol (TRA) 10 mg/kg on Von Frey filaments tactile allodynia thresholds in neuropathic model in alloxan-induced diabetic mice

**Group**	**Tactile allodynia thresholds±SEM (g)**				
	**Predose**	**2nd Week**	**4th Week**	**6th Week**	**8th Week**
NORM	7.60 ± 010	7.70 ± 0.11	8.10 ± 0.20	7.70 ± 0.13	8.10 ± 0.10
DIA + Placebo	1.40 ± 0.21	1.70 ± 0.20	1.34 ± 0.16	1.40 ± 0.10	1.44 ± 0.10
TRA 10 mg/kg	2.99 ± 0.11	23.00 ± 1.10	23.40 ± 1.20	23.50 ± 1.20	24.10 ± 1.10**
DIA + Conv	2.85 ± 0.10	6.75 ± 0.20	6.80 ± 0.20	12.75 ± 1.10	15.85 ± 1.10*
DIA + Nano	2.95 ± 0.11	7.75 ± 0.10	7.99 ± 0.20	15.960 ± 0.20	19.00 ± 0.50*

SEM: standard error of the mean * *P* < 0.05 significant from the placebo treated animals. ** *P* < 0.01 significant from the placebo treated animals.

## Conclusion

In a nutshell, 0.05 % Capsaicin nanoemulgel consisting of 8 % eucalyptus oil, 24 % S_mix_ (Tween 80: isopropyl alcohol 2:1 w/w) and 68 % water proved to enhance capsaicin skin permeation; nanoemulgel revealed an increase in the cumulative amount of permeated capsaicin by around 4 folds, and a permeation flux enhancement ratio of 1.73 was recorded when compared to the conventional gel. Interestingly, the optimized formulation ameliorated alloxan induced thermal hyperalgesia and lowered painful thresholds more significantly than the conventional gel (*P* < 0.05), hence improving its antinociceptive properties. From all these findings, it can be concluded that the selected capsaicin nanoemulgel would be a promising transdermal formulation that may alleviate DN in T1D patients.

## Ethical Issues

 Animal studies were approved by the ethics committee at Beirut Arab University, Beirut, Lebanon (approval number, 2020-A-63-P-R-0395). The conducted studies followed the guidelines of the European directives for animal experiments (2010/63/EU).

## Conflict of Interest

 There is no conflict of interest.
